# Human amniotic fluid stem cells can alleviate detrusor dysfunction caused by bladder outlet obstruction in rats

**DOI:** 10.1038/s41598-022-10640-y

**Published:** 2022-04-23

**Authors:** Ching-Chung Liang, Wen-Chu Huang, Steven W. Shaw, Yung-Hsin Huang, Tsong-Hai Lee

**Affiliations:** 1 Female Urology Section, Department of Obstetrics and Gynecology, Chang Gung Memorial Hospital Linkou Medical Center, Taoyuan, Taiwan; 2 College of Medicine, Chang Gung University, Taoyuan, Taiwan; 3 Division of Urogynecology, Department of Obstetrics and Gynecology, Mackay Memorial Hospital, Taipei, Taiwan; 4 Department of Nursing, Mackay Junior College of Medicine, Nursing, and Management, Taipei, Taiwan; 5 Division of Obstetrics, Department of Obstetrics and Gynecology, Taipei Chang Gung Memorial Hospital, Taipei, Taiwan; 6 Prenatal Cell and Gene Therapy Group, Institute for Women’s Health, University College London, London, UK; 7 Stroke Center and Department of Neurology, Chang Gung Memorial Hospital, Linkou Medical Center, No. 5, Fu-Hsing Street, Kweishan, 33333 Taoyuan Taiwan

**Keywords:** Stem cells, Diseases, Medical research, Molecular medicine, Urology

## Abstract

The present study examined whether bladder detrusor dysfunction due to partial bladder outlet obstruction (pBOO) could be improved after the treatment of human amniotic fluid stem cells (hAFSCs). 72 female rats were grouped into sham operation, pBOO, and pBOO with hAFSCs treatment (pBOO + hAFSCs) for in vitro and in vivo studies. Bladder weight, bladder wall thickness, the ratio of collagen to smooth muscle and the levels of positive CD11b/c and HIS48 cells was significantly increased after pBOO but improved after hAFSCs treatment. Cystometries showed impaired bladder function after pBOO. Protein and mRNA levels of hypoxia inducible factor-1α, CCL2, interleukin-1β, transforming growth factor-β1 (TGF-β1), connective tissue growth factor (CTGF), α-smooth muscle actin, collagen I and collagen III were increased at 2 and/or 6 weeks, but proteins and mRNA expressions of protein gene product 9.5 were decreased at 2 and 6 weeks after pBOO. These abnormalities were improved after hAFSCs treatment. The expressions of TGF-β1 and CTGF in cultured detrusor cells of pBOO rats were increased but were improved after hAFSCs treatment. The present results showed hAFSCs treatment could improve bladder detrusor dysfunction in pBOO rats, which may be related to the reduction of inflammatory and pro-fibrotic markers in detrusor muscle cells.

## Introduction

Bladder outflow obstruction (BOO) is commonly found in the elderly and can cause bladder voiding disability due to muscle damage. BOO may cause urinary retention, repeated urinary tract infections and impaired renal function. Benign prostatic hyperplasia is the leading cause of BOO in men over 50 years of age^1^. Previous surgery of urinary incontinence and pelvic organ prolapse is the most common cause of BOO in women^2,3^. BOO can cause 3 major categories of bladder dysfunctions including contractile dysfunction, unstable detrusor and abnormal compliance^4^. Approximately 24% of male BOO patients did not fully recover from bladder dysfunction after surgical intervention, because the detrusor muscle develops into underactivity after prolonged urethral obstruction^5^. Animal study also revealed a prolonged period of BOO could result in a decrease of rat bladder nerves, which could be related to detrusor underactivity after BOO^6^.

BOO is a multifactorial urological condition in which hypoxia may induce inflammatory and profibrotic response in bladder smooth muscle cells^7,8^. In addition to hypoxia, pathologic changes of BOO such as ischemia and collagen deposition also develop in bladder tissue. These changes can lead to bladder smooth muscle hypertrophy and bladder wall fibrosis, which affects the function of detrusor muscle^9,10^. To date, there is still no effective treatment for the recovery of bladder dysfunction after BOO. Experimental studies have demonstrated that adult stem cells obtained from bone marrow and adipose tissue can mediate the therapeutic effects on detrusor underactivity in animal models to prevent or reverse bladder fibrosis^11^. Besides the difficulty to obtain, adult stem cells have the limitation of restricted differentiation potential such as decreasing number with age and easy DNA damage compared with amniotic fluid-derived stem cells (AFSCs)^12^. Our previous studies have found human AFSCs (hAFSCs) could contribute to the recovery of bladder function in the situations of chronic bladder ischemia^13^ and diabetes^14^. The present study was conducted to examine the effect of hAFSCs treatment on detrusor dysfunction in rats with partial BOO (pBOO).

## Methods

### Animal preparation

Female Sprague–Dawley rats (10–12 weeks old) were maintained at 21–23 °C room temperature and 47% humidity with a 12 h light–dark cycle and given free access to standard laboratory chow and tap water ad libitum. All protocols were approved by the Institutional Ethics Committee for the Care and Use of Experimental Animals (No. 2017121812) and the Institutional Review Board of our institute (IRB no. 201701998A3). All procedures were performed in accordance with the National Institute of Health Guide for the Care and Use of Laboratory Animals (NIH Publications No. 80–23) and ARRIVE guidelines (https://arriveguidelines.org).

### Experiment design

A total of 72 rats were randomly assigned to 3 groups for in vitro and in vivo studies: (1) sham-operated rats with the injection of 0.3 mL phosphate-buffered saline (PBS) (control group); (2) pBOO rats with single injection of 0.3 mL PBS (pBOO + PBS); (3) pBOO rats with single injection of 1 × 10^6^ hAFSCs in 0.3 mL PBS (pBOO + hAFSCs). An abdominal incision was made, and either the hAFSCs or the PBS was directly injected into the submucosal layer of bladder wall using a 500-μL syringe with 26-gauge needle.

**Figure 1:**
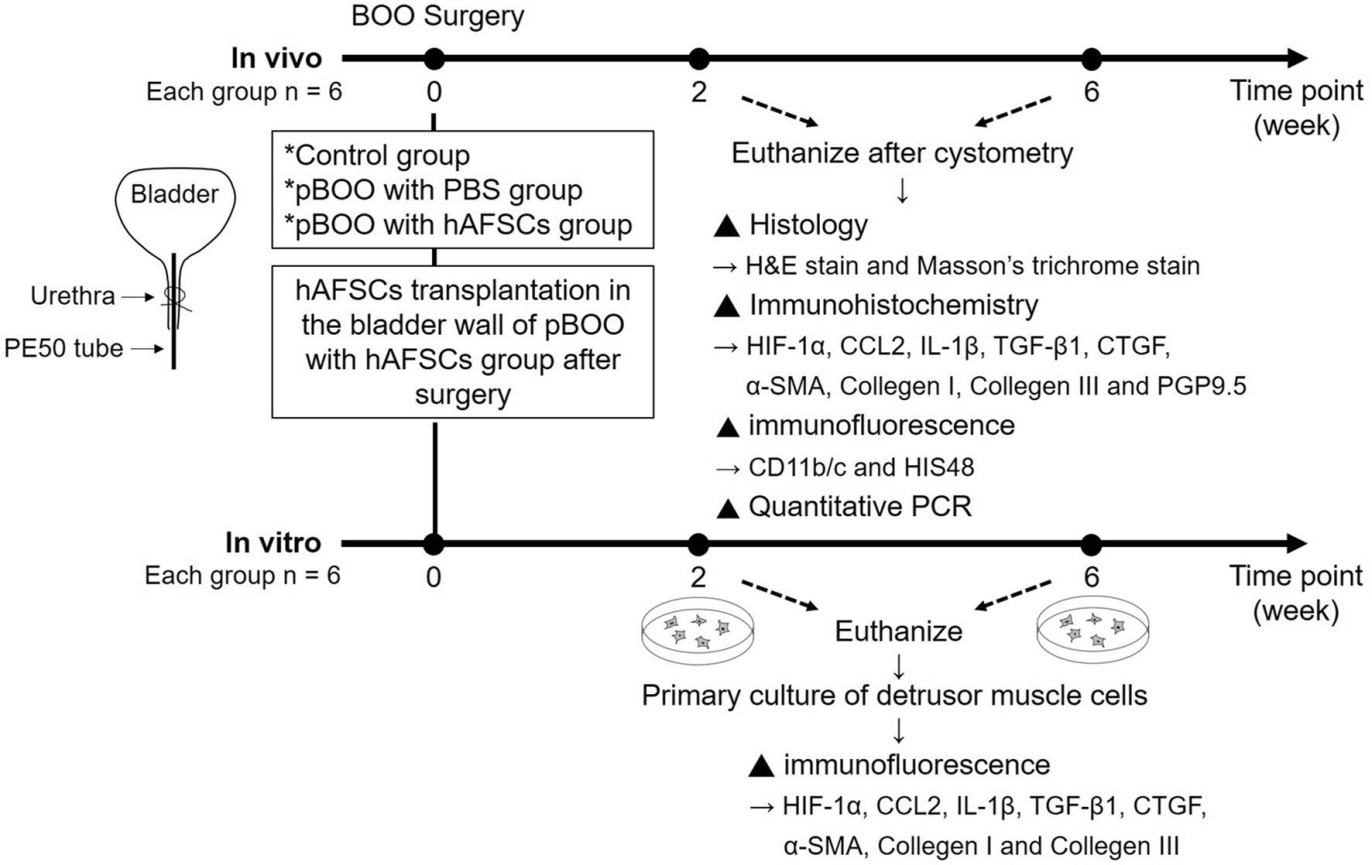
Schema of the experimental procedure. pBOO = Partial bladder outlet obstruction, PBS = Phosphate-buffered saline, hAFSCs = Human amniotic fluid stem cells, H&E = Hematoxylin and eosin, PCR = Polymerase chain reaction, HIF1α = Hypoxia inducible factor-1α, IL-1β = Interleukin-1β, TGF-β1 = Transforming growth factor-β1, CTGF = Connective tissue growth factor, α-SMA = α-smooth muscle actin, PGP9.5 = Protein gene product 9.5. Drawn by co-author, Yung-Hsin Huang.

For in vivo study, 36 rats received bladder function test using conscious cystometry at 2 and 6 weeks after pBOO (n = 6 in each time point). Animals were euthanized after cystometry, and bladders were removed and weighed. Expressions of hypoxia inducible factor-1α (HIF1α), CCL2, interleukin-1β (IL-1β), transforming growth factor-β1 (TGF-β1), connective tissue growth factor (CTGF), α-smooth muscle actin (α-SMA), collagen I, collagen III and protein gene product 9.5 (PGP9.5) in bladder were examined by immunohistochemistry and real-time polymerase chain reaction. With the same grouping method as in vivo study, another 36 rats were used in the in vitro study. The whole bladder of each rat was extirpated for detrusor muscle cell culture, and then the expressions of HIF1α, CCL2, IL-1β, TGF-β1, CTGF, α-SMA, collagen I, collagen III, CD11b/c and HIS48 [for myeloid-derived suppressor cells (MDSCs) assessment] were analyzed by immunohistochemistry. The experimental procedure is shown in Fig. 1.

### Isolation and characterization of hAFSCs for treatment

The hAFSCs were obtained from freshly collected amniotic fluid by routine amniocentesis from second-trimester healthy pregnant donors. Cells were cultured in StemPro® MSC SFM supplemented with 10% fetal bovine serum (Invitrogen, Carlsbad, CA, USA) and incubated at 37 °C with 5% CO_2_. The specific surface antigens of hAFSCs were characterized using flow cytometry according to our previous work^13–15^. The cells in culture were trypsinized and stained with phycoerythrin (PE)-conjugated antibodies against CD44, CD45, CD73, CD90, CD105 and CD117 (BD PharMingen, San Diego, CA, USA). Thereafter, the cells were analyzed using Calibur flow cytometer (Becton Dickinson, Heidelberg, Germany). Passage 6–8 hAFSCs were collected and prepared to a final concentration of 1 × 10^6^ cells/0.3 mL in PBS. In the hAFSCs-treated groups, 1 × 10^6^ collected hAFSCs were injected into 5 sites of bladder in each rat (anterior, posterior, bilateral, and dome) under inhalation anesthesia according to our previous method^14,15^. The treatment dose of hAFSCs was determined according to our previous study that used hAFSCs to treat bladder dysfunction after focal cerebral ischemia in rats^15^.

### Surgical induction of BOO

Partial BOO was made in rats through proximal urethral ligation^16^. Under 2% isoflurane inhalation, rats were placed in supine position. A suprapubic midline incision was made and abdominal cavity was opened to expose the urethrovesical junction. A polyethylene 50 tube was introduced into the urethra as the stent for urethral ligation, so that the outer diameter of catheterized urethra was about 1.0 mm. A 5–0 silk suture was loosely tied around the catheterized urethra, and the catheter was then removed shortly after. The bladder was repositioned, and the abdominal wall was closed. Control rats received temporary catheterization in the urethrovesical junction after surgical procedure without urethral ligation.

### Conscious cystometry

Two days after implantation of suprapubic catheter, animals were placed in a special metabolic cage (Med Associates Inc., St. Albans, VT, USA) for conscious cystometries at 2 and 6 weeks after sham treatment, pBOO + PBS and pBOO + hAFSCs treatment according to our previous studies^14,15^. All the cystometric parameters on 5 consecutive micturition cycles were collected for analysis. Three cystometric variables were investigated including peak voiding pressure, bladder capacity and residual volume^17^. Cystometry Analysis Version 1.05 (Catamount Research and Development, St Albans, VT, USA) was used for cystometric analysis.

### Culture of bladder smooth muscle cells

For in vitro study, rats were euthanized with an overdose of isoflurane. The tissue of bladder body was placed on ice, and then the mucosa and the serosa on detrusor were quickly trimmed off with scissors. The smooth muscle layer was incubated at 37 °C for 30 min in PBS(-) containing 0.2% trypsin (Gibco, Thermo Fisher Scientific, USA) in a shaking incubator^18^. The smooth muscle layers were finely minced with scissors and allowed to suspend in RPMI-1640 medium (Gibco, Thermo Fisher Scientific, USA) containing 0.1% collagenase (Gibco, Thermo Fisher Scientific, USA). After digestion, cell suspension was incubated at 37 °C for 30 min, followed by centrifugation at 250 × *g* to remove supernatant. Pellet was re-suspended with RPMI-1640 containing 10% fetal bovine serum and centrifugated at 250 × g. Supernatant (I) was collected, and pellet was re-suspended with RPMI-1640 containing 10% fetal bovine serum and then centrifugated at 125 × g. Supernatant (II) and pellet contaminating tissues including fibroblasts, intimal cells and tissue debris were also collected. The supernatant fraction which contained smooth muscle cells was collected. Then, the pure smooth muscle cells were cultured on dish containing RPMI-1640 supplemented with 10% fetal bovine serum in a humidified atmosphere of 95% air and 5% CO_2_. The culture medium was changed every 3 days. At 7 days, the cultured smooth muscle cells of rat bladder body were immunostained with anti-α-actin antibody.

### Tissue preparation

After cystometries were performed, all rats were euthanized with an overdose of isoflurane. The dissected bladder walls were fixed in optimal cutting temperature (OCT) compound, frozen in liquid nitrogen, and stored at − 80 °C. The bladders were then subjected to cryosection to yield tissue sections of 12-μm thickness at − 20 °C, and the sections were transferred to glass microscope slides coated with silane (Muto Pure Chemical, Tokyo, Japan).

### Histology assessment

Hematoxylin and Eosin (H&E) staining was performed for the measurements of bladder wall thickness, and Masson's trichrome staining was performed for histopathological observation. The bladder wall thickness was measured at the 4 ends of a cross in 3 whole bladder sections separated in 1 mm for each rat. The smooth muscle and the connective tissue were stained in red and blue, respectively. The percentage of bladder tissue affected by fibrosis was determined by calculating the ratio of collagen area to smooth muscle area. The bladder wall thickness and areas for connective tissue and total tissue were quantitated using Image-Pro Plus Software (Media Cybernetics, Silver Spring, MD, USA) under Olympus BX-51 microscope and compared among the groups.

### Immunohistochemical and immunofluorescent assessment

The dissected bladders were fixed in OCT compound, frozen in liquid nitrogen, and stored at − 80 °C. The bladders were then subjected to cryosection yielding sections of 12-μm thickness at − 20 °C, and the sections were transferred to glass microscope slides coated with silane (Muto Pure Chemical, Tokyo, Japan).

Bladder sections were immunostained against HIF1α, CCL2, IL-1β, TGF-β1, CTGF, α-SMA, collagen I, collagen III and PGP9.5 using the avidin–biotin peroxidase method, and against CD11b/c and HIS48 using the immunofluorescent method. The catalogue number, isotype, dilution concentration, host species and manufacturer of each antibody were summarized in Supplementary Table S1. First, fresh-frozen sections were fixed for 10 min in acetone (Honeywell, Germany) or 4% paraformaldehyde (PFA, Merck Millipore, USA). Acetone was used for HIF-1α, CCL2, IL-1β, TGF-β1, CTGF and PGP9.5 antibodies and 4% PFA for α-SMA, collagen I, collagen III, CD11b/c and HIS48 antibodies. After blocking with Dako REAL peroxidase blocking solution (DAKO Corp, Carpinteria, CA, USA) against HIF1α, CCL2, IL-1β, TGF-β1, CTGF, α-SMA, collagen I, collagen III and PGP9.5 for 20 min and with 1% bovine serum albumin (BSA, Thermo Fisher Scientific, USA) against CD11b/c and HIS48 for 30 min, sections were washed and incubated with primary antibody for 20 h at 4 °C. Sections were then washed and incubated for 1 h with biotinylated secondary antibodies (1:200, Vector Laboratories, Burlingame, CA, USA) or Alexa-fluor 488 (1:250, Invitrogen, Grand Island, NY, USA). Staining was developed with 3, 3’-diaminobenzidine (DAB, Thermo Fisher Scientific, USA) plus hydrogen peroxide as the chromogen and counterstained with hematoxylin for HIF1α, CCL2, IL-1β, TGF-β1, CTGF, α-SMA, collagen I, collagen III and PGP9.5. In addition, nuclear staining using CD11b/c and HIS48 was performed with 4', 6-diamidino-2-phenylindole (DAPI, BIOTOOLS CO., LTD, Taipei, Taiwan). Sections from the three experimental groups were put on the same slide during staining to keep same incubation time for the antibodies and chromogen.

Immunofluorescence against HIF1α, CCL2, IL-1β, TGF-β1, CTGF, α-SMA, collagen I, collagen III and PGP9.5 was done on detrusor smooth muscle cells. Detrusor smooth muscle cells of 2–3 passages were collected, plated at an appropriate density and attached to a 12-well plate. Cells were fixed in 4% paraformaldehyde for 5 min and then rinsed 3 times with PBS. After incubation with 1% BSA blocking agent and 0.1% Triton-X 100 (Sigma-Aldrich, USA), cells were washed and incubated for 20 h at 4 °C with primary antibodies, and then incubated for 1 h with secondary antibody, Alexa-fluor 488 (1:250, Invitrogen, Grand Island, NY, USA). Nuclear staining was performed with 4', 6-diamidino-2-phenylindole (DAPI).

Immunoreactivity was analyzed using Image-Pro Plus Software (Media Cybernetics, Silver Spring, MD, USA) under Olympus BX-51 microscope. The ratio of immunoreactivity level of pBOO rats with or without hAFSCs treatment to that of sham rats was determined for all the reactions.

### Quantitative reverse transcription PCR of bladder

Real-time PCR was carried out according to the manufacturer’s protocol. Total RNAs were prepared using a Trizol reagent (Invitrogen, Carlsbad, CA, USA) and incubated in reverse transcription mixture at 25 °C for 5 min, 50 °C for 1 h, 70 °C for 15 min; finally, the tubes were cooled to 4 °C for 5 min. Gene expression for HIF1α, CCL2, IL-1β, TGF-β1, CTGF, α-SMA, collagen I, collagen III and PGP9.5 in the bladder tissue was analyzed by real-time PCR using inventoried TaqMan assays from Applied Biosystems (Life Technologies, Grand Island, NY, USA). The assays codes of HIF1α (Rn01472831-m1), CCL2 (Rn00580555-m1), IL-1β (Rn00580432-m1), TGF-β1 (Rn00572010-m1), CTGF (Rn01537279-g1), α-SMA (Rn01759928-g1), collagen I (Rn01463848-m1), collagen III (Rn01437681-m1) and PGP9.5 (Rn00568258-m1) were obtained from manufacturer (Applied Biosystems, Oster City, CA, USA). GAPDH (assay code Rs99999916-s1) was used as an endogenous control to allow for quantification of relative gene expression. Thermal cycling and fluorescence detection were performed using an ABI Prism 7900HT Sequence Detection System (Applied Biosystems, Oster City, CA, USA). PCR conditions were 50 °C for 2 min, 95 °C for 10 min, followed by 40 cycles at 95 °C for 15 s and 60 °C for one min. The data were calculated using the 2[-Delta Delta C(T)] method^19^. A ratio of the mRNA level of pBOO rats with or without hAFSCs treatment to that of sham rats was determined. The values were summated and expressed as mean ± SD and were compared statistically among the groups and among different time points in each group.

### Statistical analysis

Data were analyzed with Prism 5 (GraphPad Software, San Diego, CA, USA) and expressed as mean ± SD for continuous variables. Continuous data were compared among the groups by using one-way analysis of variance. The Tukey–Kramer test was used for post-hoc comparisons. To evaluate the effect of hAFSCs among groups, Chi-Square test was performed with Fisher’s Exact Test. Probability values of < 0.05 were considered to be statistically significant.

## Results

### Bladder weights of pBOO rats improved after hAFSCs treatment

**Figure 2:**
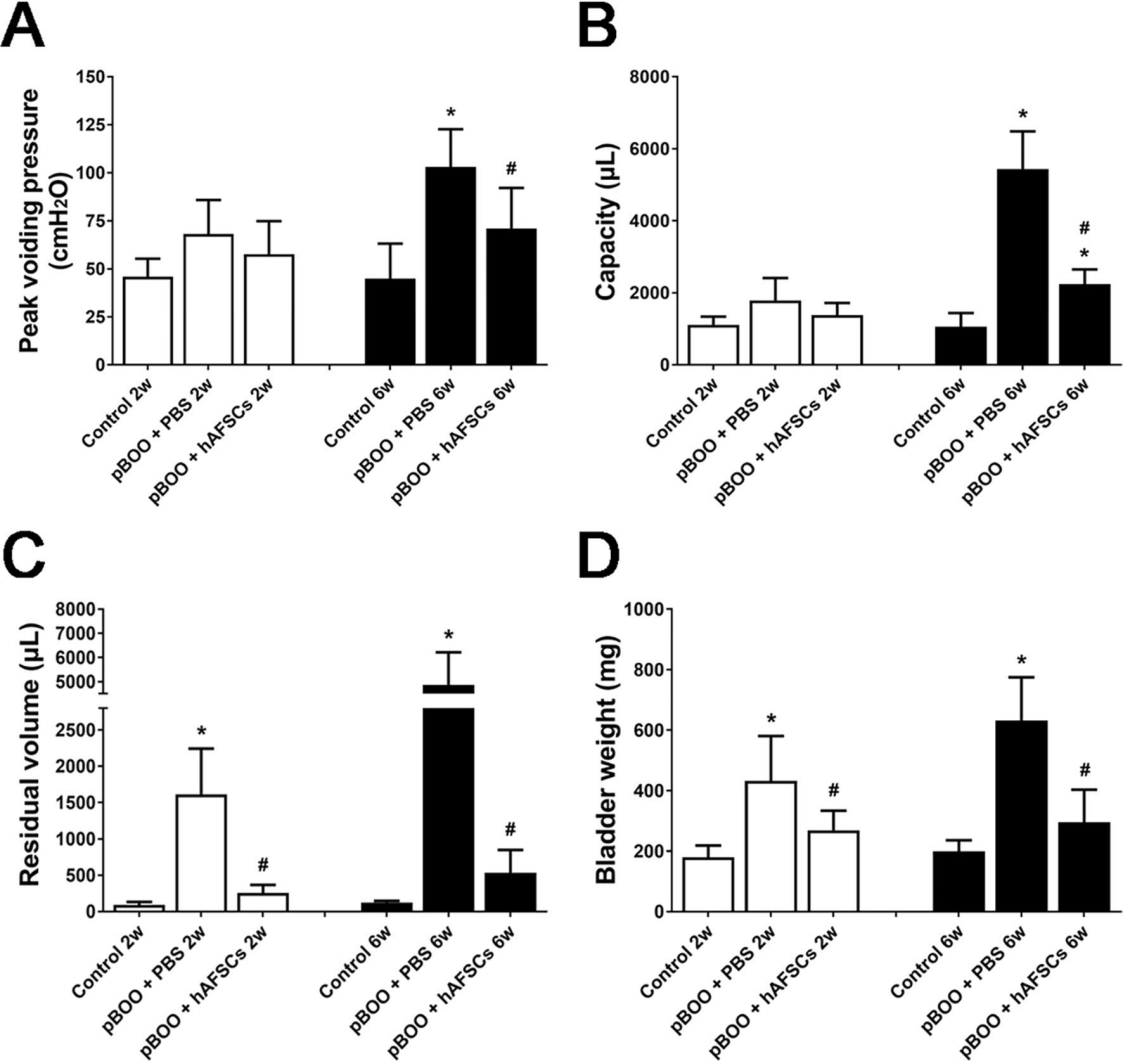
Cystometric results (**A**–**C**) and bladder weight (**D**) in the control, pBOO + PBS and pBOO + hAFSCs treatment at 2 and 6 weeks after pBOO are presented. Cystometric variables include peak voiding pressure (**A**), bladder capacity (**B**) and residual volume (**C**). Bladder dysfunction and bladder weight in the pBOO rats can be improved after hAFSCs treatment. *: *P* < 0.05 vs. control, #: *P* < 0.05 vs. pBOO + PBS. N = 6 in each time point.

Compared with sham-operated controls, pBOO group had increased bladder weight significantly at 2 and 6 weeks after pBOO. However, hAFSC treatment significantly recovered bladder weight in pBOO rats at 2 and 6 weeks (Fig. 2D).

### Bladder dysfunctions of pBOO rats improved after hAFSCs treatment

The peak voiding pressure and bladder capacity of pBOO rats were not increased at 2 weeks but were significantly increased compared with control rats at 6 weeks. The residual volume was also significantly increased at 2 and 6 weeks compared with the control group. These parameters of bladder dysfunction were improved after hAFSCs treatment (Fig. 2).

### Bladder wall thickness and the ratio of collagen to smooth muscle improved after hAFSCs treatment

**Figure 3:**
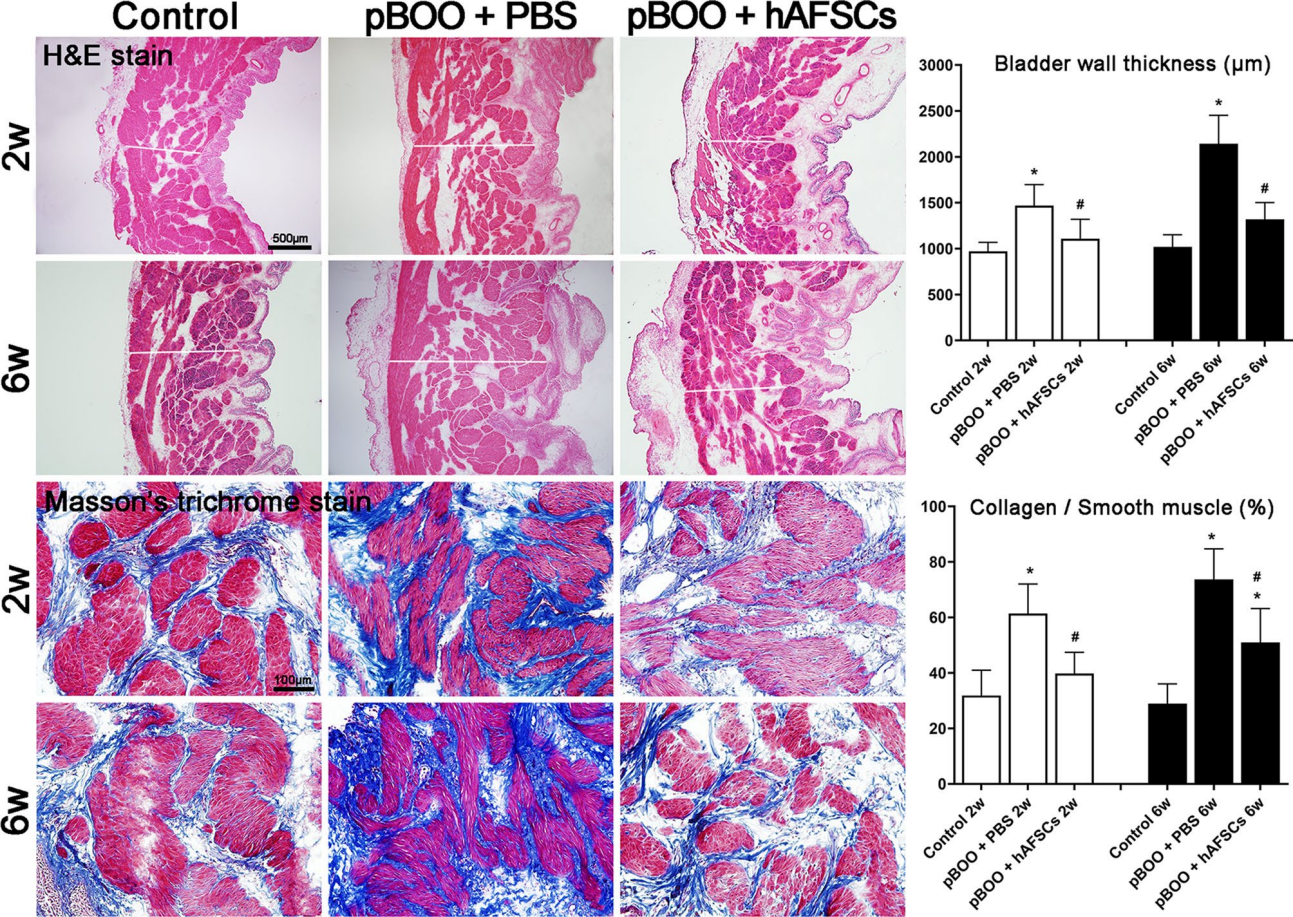
Hematoxylin–eosin and Masson's trichrome staining of the bladder sections with quantitation of bladder wall thickness and ratio of collagen to smooth muscle. Bladder wall is thickened with detrusor muscle hypertrophy at 2 and 6 weeks after surgery in pBOO rats compared with control groups and the rats after hAFSCs treatment. Representative immunohistochemical staining shows the collagen with blue staining and the muscle with red staining. The collagen deposition increases in the pBOO group and recovers after hAFSCs treatment. *:*P* < 0.05 vs. control, #: *P* < 0.05 vs. pBOO + PBS. N = 6 in each time point. Bar indicates 500 μm in Hematoxylin–eosin staining and 100 μm in Masson’s Trichrome staining, respectively. The bladder wall thickness was measured at the 4 ends of a cross in 3 whole bladder sections separated in 1 mm for each rat. The horizontal white line in each panel of Hematoxylin–eosin staining indicates the location of measuring bladder wall thickness.

Compared with the control group, the bladder wall thickness in pBOO group was significantly increased at 2 and 6 weeks, mainly due to hypertrophy of the detrusor muscle layer. Compared with the pBOO group, the bladder wall thickness of hAFSCs treatment group was significantly reduced at 2 and 6 weeks after treatment (Fig. 3). Compared with pBOO rats, hAFSCs treatment reduced the deposition of collagen fibers in the lamina propria and detrusor muscle layer. Compared with control group, the ratio of collagen to smooth muscle was significantly decreased in the hAFSCs rats at 2 and 6 weeks after treatment. However, unlike that at 2 weeks, the ratio of collagen to smooth muscle did not return to near the control levels at 6 weeks after hAFSCs treatment (Fig. 3).

### Expressions of HIF1α, CCL2, IL-1β, TGF-β1, CTGF, α-SMA, collagen I, collagen III and PGP9.5 improved after hAFSCs treatment

**Figure 4:**
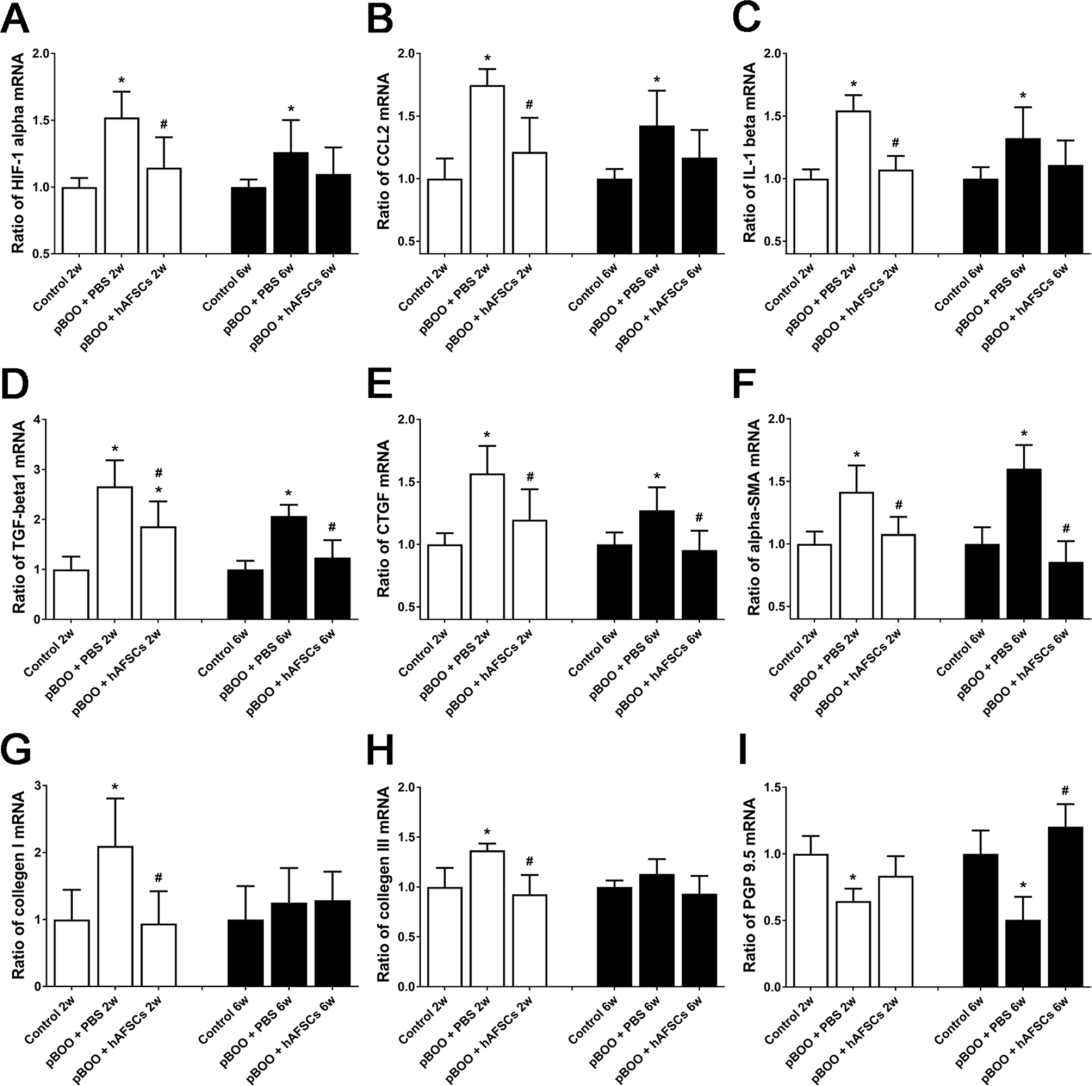
Relative mRNA expressions of hypoxia inducible factor-1α (HIF1α, **A**), CCL2 (**B**), interleukin-1β (IL-1β, **C**), transforming growth factor-β1 (TGF-β1, **D**), connective tissue growth factor (CTGF, **E**), α-smooth muscle actin (α-SMA, **F**), collagen I (**G**), collagen III (**H**) and protein gene product 9.5 (PGP9.5, I) in the bladder of control, pBOO + PBS and pBOO + hAFSCs treatment. After hAFSCs treatment, the mRNA expressions of CTGF and α-SMA are improved at 2 and 6 weeks after pBOO, but HIF1α, CCL2, IL-1β, TGF-β1, collagen I and collagen III are improved only at 2 weeks after pBOO. The mRNA levels of PGP9.5 are improved after hAFSCs treatment only at 6 weeks after pBOO. *: *P* < 0.05 vs. control, #: *P* < 0.05 vs. pBOO + PBS. N = 6 in each time point.

**Figure 5:**
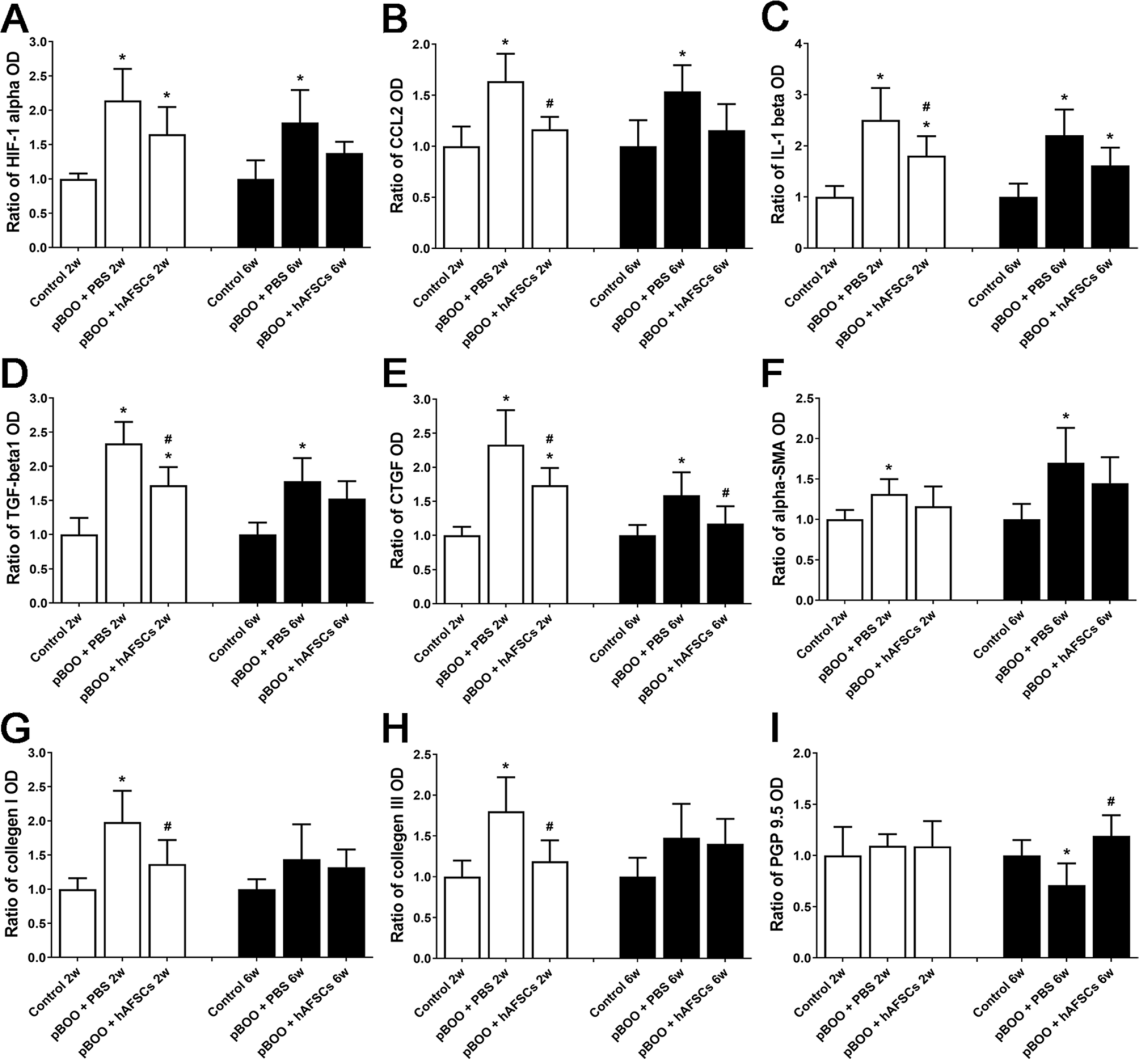
Expressions of the immunoreactivities of hypoxia inducible factor-1α (HIF1α, **A**), CCL2 (**B**), interleukin-1β (IL-1β, **C**), transforming growth factor-β1 (TGF-β1, **D**), connective tissue growth factor (CTGF, E), α-smooth muscle actin (α-SMA, F), collagen I (**G**), collagen III (**H**) and protein gene product 9.5 (PGP9.5, **I**) in the bladder of control, pBOO + PBS and pBOO + hAFSCs treatment. After hAFSCs treatment, the immunoreactivities of CTGF and α-SMA are improved at 2 and 6 weeks after pBOO, but HIF1α, CCL2, IL-1β, TGF-β1, collagen I and collagen III are improved only at 2 weeks after pBOO. The immunoreactivities of PGP9.5 are improved after hAFSCs treatment only at 6 weeks after pBOO. *: *P* < 0.05 vs. control, #: *P* < 0.05 vs. pBOO + PBS. N = 6 in each time point.

The immunoreactivities and mRNA levels of HIF1α, CCL2, IL-1β, TGF-β1, CTGF, α-SMA, collagen I and collagen III in pBOO rats were increased at 2 and/or 6 weeks, but PGP9.5 were decreased at 2 and 6 weeks after pBOO. After hAFSCs treatment, the immunoreactivities and mRNA levels of CTGF and α-SMA were improved at 2 and 6 weeks, HIF1α, CCL2, IL-1β, TGF-β1, collagen I and collagen III were improved only at 2 weeks, and those of PGP9.5 were improved at 6 weeks (Figs. 4 and 5, and Supplementary Fig. 1).

### Level of MDSCs decreased after hAFSCs treatment

**Figure 6:**
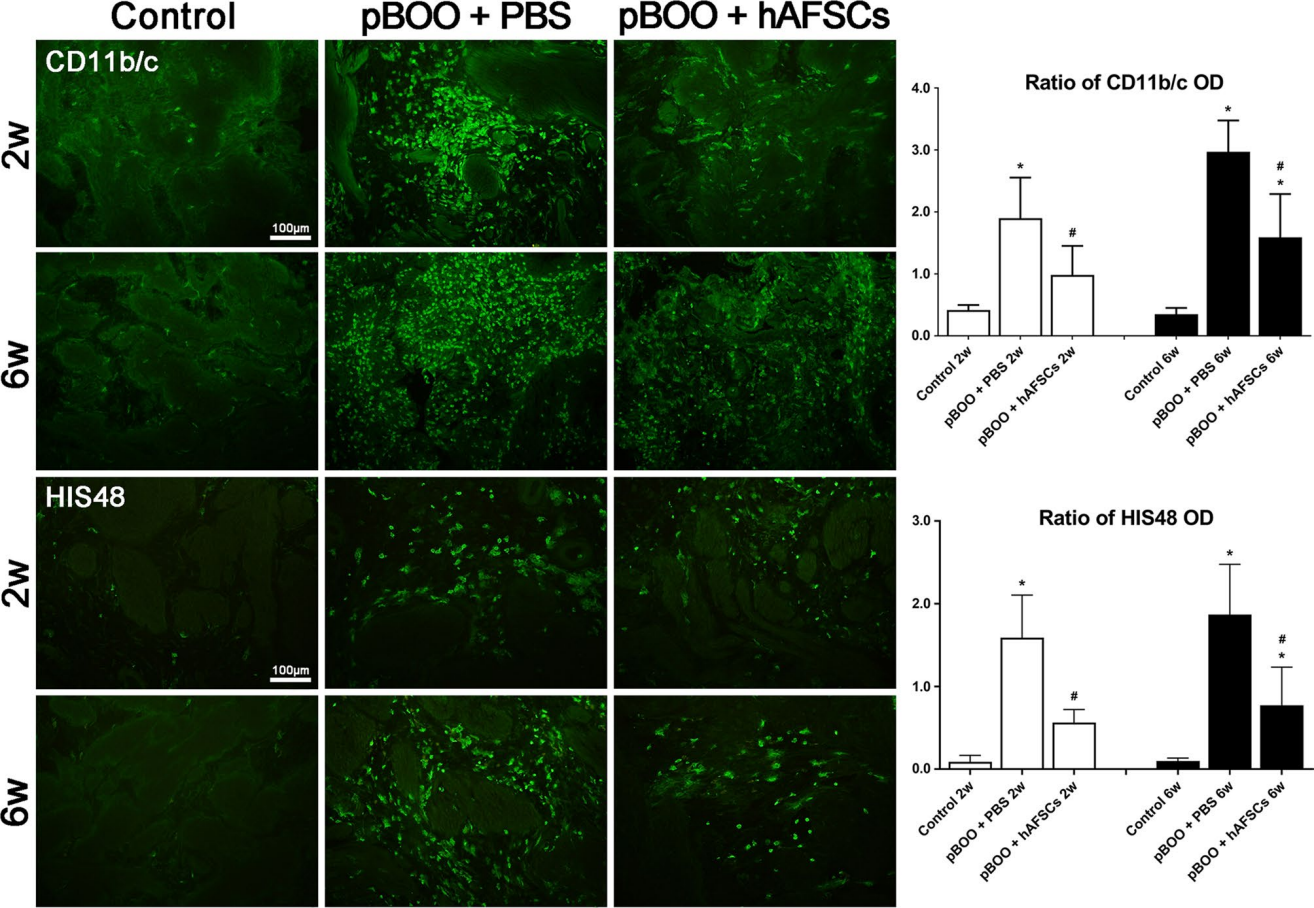
Myeloid-derived suppressor cells (MDSCs) are stained positive for both CD11b/c and HIS48 in bladder at different time points after pBOO and hAFSCs treatment. The levels of positive CD11b/c and HIS48 stained MDSCs are increased in the pBOO group compared with the control group and decreased at 2 and 6 weeks after hAFSCs treatment. *: *P* < 0.05 vs. control, #: *P* < 0.05 vs. pBOO + PBS. N = 6 in each time point. Bar indicates 100 μm.

Compared with the control group, the levels of positive CD11b/c and HIS48 stained MDSCs were significantly increased in the pBOO group but decreased at 2 and 6 weeks after hAFSCs treatment. However, unlike that at 2 weeks, the levels of positive CD11b/c and HIS48 stained MDSCs did not return to near the level of control rats at 6 weeks after hAFSCs treatment (Fig. 6).

### TGF-β1 and CTGF expression in cultured detrusor cells of pBOO rats improved after hAFSCs treatment

**Figure 7:**
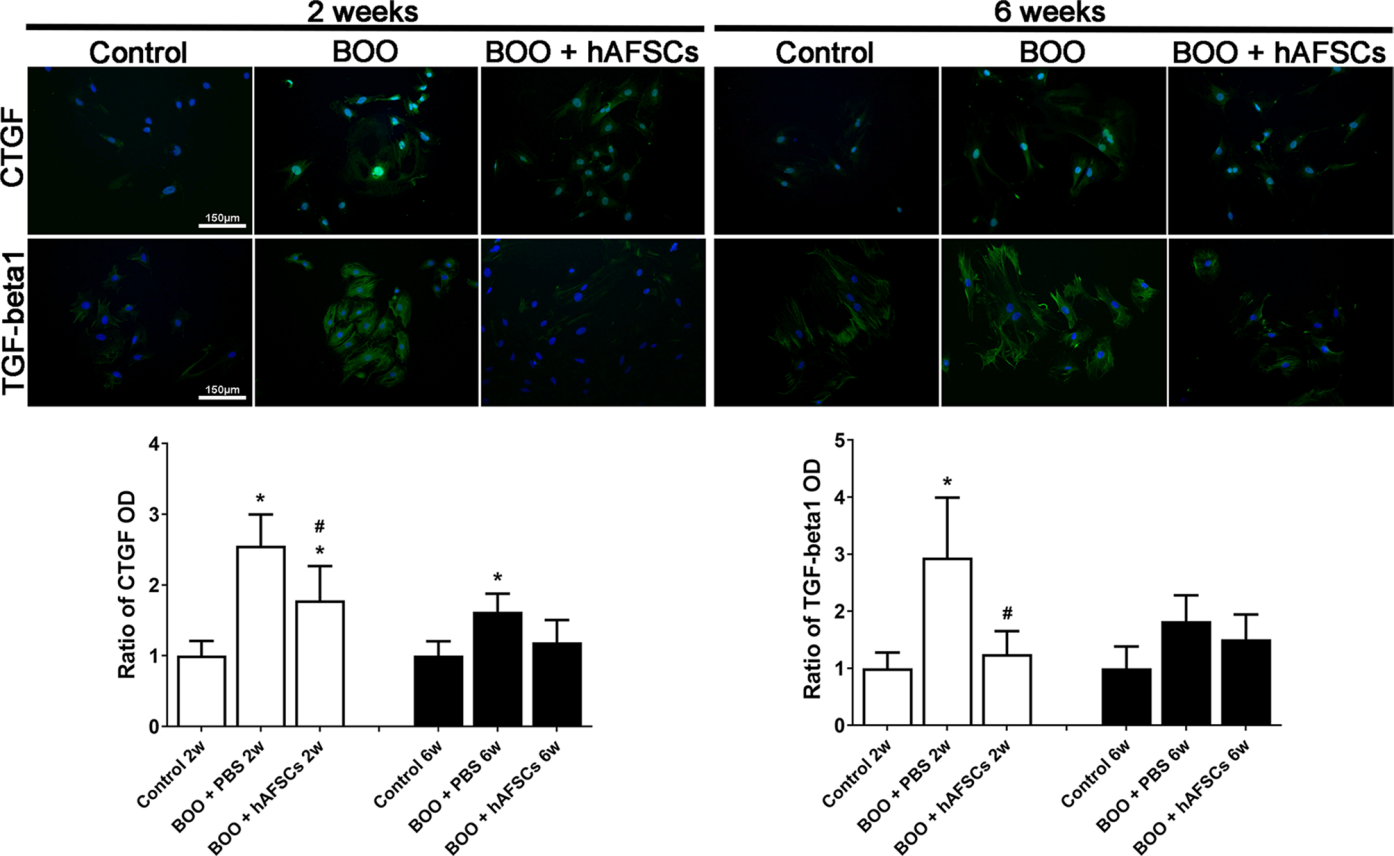
Temporal immunofluorescent expressions of CTGF and TGF-β1 in cultured detrusor cells of control, pBOO + PBS and pBOO + hAFSCs. Compared with the control, cultured detrusor cells of pBOO rat have higher CTGF immunoreactivity at 2 and 6 weeks after pBOO, and higher TGF-β1 immunoreactivity at 2 weeks after pBOO. All these changes can be improved after hAFSCs treatment. *: *P* < 0.05 vs. control, #: *P* < 0.05 vs. pBOO + PBS. N = 6 in each time point. Bar indicates 150 μm.

Compared with control rats, cultured detrusor cells of pBOO rat had higher CTGF immunoreactivity at 2 and 6 weeks, and higher TGF-β1 immunoreactivity at 2 weeks. All these changes could be improved after hAFSCs treatment (Fig. 7).

## Discussion

In pBOO rats, we demonstrated that the immunoreactivities of TGF-β1 and CTGF in cultured detrusor cells were increased at 2 weeks and then decreased at 6 weeks after pBOO. This trend of change is consistent with that of intact bladder tissue. However, the expression of other inflammatory and pro-fibrosis markers in detrusor muscle cells did not show a significant up-regulation like intact bladder tissue. Our study suggests that the results in cultured cells did not necessarily reveal similar expression pattern to those of in vivo study.

Our results indicated that in pBOO rats, hAFSCs treatment appeared to be associated with restoration of normal bladder function. At 2 and/or 6 weeks after pBOO, bladder weight and bladder dysfunction such as peak voiding pressure, bladder capacity and residual volume could be improved after hAFSCs treatment. The improvement of bladder function after hAFSCs treatment is correlated with decreased detrusor muscle hypertrophy and fibrosis. After hAFSCs treatment, the abnormalities of HIF1α, CCL2, IL-1β, TGF-β1, CTGF, α-SMA, collagen I, collagen III and PGP9.5 were improved at 2 and/or 6 weeks after pBOO. In addition, after hAFSCs treatment, the immunoreactivities of CTGF and TGF-β1 in cultured detrusor cells of pBOO rats were also improved.

The bladder weight increased after pBOO surgery, which may be due to the increase of bladder wall thickness and muscle layer, since bladder wall thickness and muscular hypertrophy could represent the total bladder mass and is suggested being able to predict BOO^16^. In the present study, the bladder wall thickness in pBOO group was increased significantly at 2 and 6 weeks but reduced after hAFSCs treatment. Previous studies showed that pBOO could be caused by collagen deposit in bladder wall and eventually resulted in bladder fibrosis, which may adversely affect detrusor function and bladder capacity to empty^20,21^. In the present study, compared with control rats, pBOO rats had a higher residual volume, but peak voiding pressure and bladder capacity did not increase significantly at 2 weeks. At 6 weeks after pBOO, the residual volume of pBOO rats increased markedly, leading to a significant increase in peak voiding pressure and bladder capacity. Similar to the present results, Lee et al. found that maximal voiding pressure and residual volume increased at 6 weeks after BOO^22^. Metcalfe et al. ^10^ reported that the early urodynamics of pBOO rats revealed an increase in bladder capacity with normal pressure but deteriorated into the bladders with small capacity and high pressure at 8 weeks after pBOO. By 13 weeks, the bladders became decompensated due to very high pressure, and there was a significant decrease in bladder capacity^10^. Subsequent loss of bladder muscle contractility due to BOO can progress to detrusor underactivity.

Previous studies have pointed out that the development of pBOO-induced bladder dysfunction carries a sequential process of inflammation, hypertrophy and final fibrosis^7,10^. After pBOO, bladder smooth muscle may have inflammatory and hypoxic response initially, which then result in detrusor muscle hypertrophy and increased intravesical pressures^10^. The present study shows that the immunoreactivities and mRNA levels of inflammatory and hypoxia factors such as HIF1α, CCL2, IL-1β and TGF-β1 in pBOO rats are up-regulated at 2 weeks and then slowly decreased at 6 weeks after pBOO. Previous experimental studies have demonstrated pBOO-induced hypoxia significantly increased HIF1α mRNA expression and promoted other pro-inflammatory cytokines, TGF-β1, IL-1β and CCL2^10,23–27^. Increased TGF-β1 may activate TGFβ-SMAD signaling pathway^28^, and activating TGF-β/SMAD signaling pathway may promote bladder fibrosis secondary to pBOO^29^. As activation of TGFβ-SMAD pathway could also cause the downstream activation of CTGF to modulate cell growth and collagen synthesis^7^ and tissue hypoxia could induce the expression and secretion of CTGF^30^, it is possible CTGF can act in the mechanism of tissue damage caused by hypoxia.

CTGF and TGF-β are reported to play an important role in the fibrosis of the bladder and other organ systems^10,31,32^. Metcalfe et al. reported that the mRNA levels of CTGF, TGF-β1 and collagen increased significantly in the first 2 weeks of pBOO and decreased to the same baseline levels as pBOO at 13 weeks^10^. The present study also demonstrated that at 2 weeks after pBOO, there was an up-regulation of pro-fibrotic mediators such as CTGF, α-SMA, collagen I and collagen III, but CTGF, collagen I and collagen III were down-regulated at 6 weeks after pBOO. In addition, the expression of α-SMA increased with the progression of pBOO. Previous study showed that up-regulation of CTGF and TGF-β1 were consistent with the subsequent increase in α-SMA. Our results support the findings of previous studies that after pBOO, collagen levels increased at 2 weeks, but the effects of increased collagen and extracellular matrix may not appear until after prolonged obstruction^10,33^. Collagen I and collagen III are the key interstitial collagens most commonly found in obstructive bladder fibrosis^10,29,33^. Denervation of the bladder is an adverse consequence of pBOO^34^. In the present study, the immunoreactivity and mRNA of PGP9.5, a pan-neuronal marker, were down-regulated more severely at 6 weeks after pBOO than that at 2 weeks.

Some preclinical studies have demonstrated that bladder wall or intravenous injection of mesenchymal stem cells obtained from bone marrow or adipose tissue can improve bladder dysfunction in model of pBOO^22,23,25,35^. In the present study, bladder weight and bladder dysfunction such as peak voiding pressure, bladder capacity and residual volume were improved at 2 weeks and/or 6 weeks after hAFSCs injected into the bladder wall. Lee et al. have indicated that transplanting human bone marrow-derived mesenchymal stem cells (BMSCs) labeled with nanoparticles into the bladder can inhibit bladder fibrosis and induce improvements in bladder dysfunction^22^. Similar to our results, previous studies revealed that the expression of collagen and TGF-β protein increased at 2 and 6 weeks after pBOO and recovered after human BMSCs treatment with or without overexpressing hepatocyte growth factor^22,35^. Intravenous administration of rat BMSCs in pBOO rats have demonstrated a significant decrease in bladder capacity and mRNA levels of TGF-β1, HIF1α and other inflammatory and pro-fibrosis markers after 2 and/or 4 weeks^23,25^. In the present study, we found that pBOO-induced inflammation led to an increase in IL-1β, which was inferred to enhance immunosuppression by inducing MDSCs. Compared with the control groups, the expression of IL-1β and the level of positive CD11b/c and HIS48 stained MDSCs were significantly decreased after hAFSCs treatment. Previous studies have shown that inflammation-induced MDSCs facilitate tumor progression through IL-1β to suppress immune responses in murine models^36,37^. The levels of circulating MDSCs with positive double staining for CD11b/c and HIS48 have been found to be significantly increased in pBOO rats compared with control rats and returned to normal after reversal of obstruction^38^. Furthermore, our in vitro analysis demonstrated that the expressions of TGF-β1 and CTGF increased in cultured detrusor cells of pBOO rats but decreased after hAFSCs treatment. The aforementioned results regarding the significant down-regulation of inflammatory and pro-fibrotic mediators indicate that hAFSCs treatment can improve bladder dysfunction and reduce collagen deposition in pBOO rat, which may act through anti-inflammatory and anti-fibrotic reactions. Regarding the therapeutic mechanism of hAFSCs on pBOO, we hypothesized that pBOO-induced hypoxia and inflammation could lead to an increase in pro-inflammatory chemokine CCL2, which would recruit hAFSCs to the bladder, thereby triggering anti-inflammatory and anti-fibrotic responses. As a result, hAFSCs could downregulate TGF-β1 and inactivate the TGFβ-SMAD pathway in pBOO rats, then inhibiting the formation of bladder fibrosis. On the other hand, hAFSCs could also downregulate CTGF secretion and prevent collagen deposition on bladder.

Although preclinical evidence suggests that mesenchymal stem cells have a potential therapeutic role in pBOO-related bladder dysfunction, only few clinical studies are currently undergoing^39^. The only clinical trial using autologous stem cells for the treatment of BOO/detrusor underactivity has involved the use of autologous muscle-derived cells (AMDC). Levanovich et al. have reported a case of cell therapy using AMDC to treat detrusor underactivity^40^. At one year follow-up, the maximum cystometric capacity was found to improve from 844 to 663 ml, and no study-related adverse events or side effects could be observed. Diokno et al. performed transurethral injection of AMDC in 15 women with detrusor underactivity, and among them, 12 received a second injection^41^. Interim analysis up to 12 months showed encouraging improvement in bladder function, and no serious treatment-related adverse effects occurred. Since hAFSCs can be obtained from amniotic fluid, grow easily in culture and appear phenotypically and genetically stable, our and previous studies have suggested hAFSCs can act as a novel source for cell transplantation therapy better than other adult stem cells^12–15,17,42^.

To our knowledge, our study is the first report of hAFSCs treatment to the bladder in a murine pBOO model. In addition, no previous studies have used detrusor cell cultures to demonstrate whether cells cultured after treatment with hAFSCs had an expression pattern similar to the in vivo study. However, the present study has limitations. First, the present study used single treatment of hAFSCs at one fixed density. It is possible that repeated treatment with a higher density could obtain better effects on bladder dysfunction induced by pBOO. Second, functional and morphological alterations were examined in the pBOO bladders at 2 and 6 weeks after hAFSCs treatment. It is possible that there would be different results if cystometric analysis could be studied at a longer period after hAFSCs treatment. Third, the present study did not examine the cellular differentiation of hAFSCs after treatment into bladders. However, our previous study found that the number of hAFSCs was decreased to nearly zero at 14 days after single injection into spinal cord injured site, but bladder dysfunction could still be improved at 28 days after hAFSCs treatment^42^. This result may suggest that the effect of hAFSCs treatment may sustain for a longer period to help with the recovery of bladder dysfunction. Also, it is likely that the functional improvement of bladder following spinal cord injury may be due to multimodal actions including neurogenesis caused by hAFSCs treatment^42^. Fourth, the present study did not examine the presence of immunosuppression. However, our previous data have revealed that the hAFSCs injection in animal studies was not found to develop any immune rejection against transplanted tissue^13–15,17,42^.

## Conclusion

Our results show that pBOO may induce inflammatory and hypoxic response, which causes hypertrophy, fibrosis and denervation of the bladder wall in rat. The improvement of bladder dysfunction in pBOO rats by hAFSCs treatment may be associated with anti-inflammatory and anti-fibrotic reactions.

## Informed consent

In this article, animal model was used for study. All protocols were approved by the Institutional Ethics Committee for the Care and Use of Experimental Animals (No. 2017121812) and the Institutional Review Board of our institute (IRB no. 201701998A3). A total of 5 human subjects donated amniotic fluid for stem cell treatment, and their informed consents were obtained.

## Supplementary Information

Supplementary Information 1.

Supplementary Information 2.

Supplementary Information 3.

## Data Availability

The datasets used and/or analysed during the current study are available from the corresponding author on reasonable request.

## References

[1] Wei JT, Calhoun E, Jacobsen SJ (2008). Urologic diseases in america project: Benign prostatic hyperplasia. J. Urol..

[2] Groutz A, Blaivas JG, Chaikin DC (2000). Bladder outlet obstruction in women: Definition and characteristics. Neurourol. Urodyn..

[3] Bradley CS, Rovner ES (2004). Urodynamically defined stress urinary incontinence and bladder outlet obstruction coexist in women. J. Urol..

[4] Yu G, Bo S, Xiyu J, Enqing X (2003). Effect of bladder outlet obstruction on detrusor smooth muscle cell: An in vitro study. J. Surg. Res..

[5] Rodrigues P, Lucon AM, Freire GC, Arap S (2001). Urodynamic pressure flow studies can predict the clinical outcome after transurethral prostatic resection. J. Urol..

[6] Tyagi P (2014). Pathophysiology and animal modeling of underactive bladder. Int. Urol. Nephrol..

[7] Wiafe B (2017). Hypoxia-increased expression of genes involved in inflammation, dedifferentiation, pro-fibrosis, and extracellular matrix remodeling of human bladder smooth muscle cells. In Vitro Cell Dev. Biol. Anim..

[8] Wiafe B, Adesida A, Churchill T, Metcalfe P (2018). Mesenchymal stem cells inhibit hypoxia-induced inflammatory and fibrotic pathways in bladder smooth muscle cells. World J. Urol..

[9] Komninos C, Mitsogiannis I (2014). Obstruction-induced alterations within the urinary bladder and their role in the pathophysiology of lower urinary tract symptomatology. Can. Urol. Assoc. J..

[10] Metcalfe PD (2010). Bladder outlet obstruction: Progression from inflammation to fibrosis. BJU Int..

[11] Chai TC, Kudze T (2017). New therapeutic directions to treat underactive bladder. Investig. Clin. Urol..

[12] De Coppi P (2007). Isolation of amniotic stem cell lines with potential for therapy. Nat. Biotechnol..

[13] Liang CC, Shaw SS, Lin YH, Lee TH (2018). Amniotic fluid stem cells ameliorate bladder dysfunction induced by chronic bladder ischemia in rat. Neurourol. Urodyn..

[14] Liang CC, Shaw SS, Huang YH, Lin YH, Lee TH (2018). Improvement in bladder dysfunction after bladder transplantation of amniotic fluid stem cells in diabetic rats. Sci. Rep..

[15] Liang CC, Shaw SW, Huang YH, Lin YH, Lee TH (2017). Bladder transplantation of amniotic fluid stem cell may ameliorate bladder dysfunction after focal cerebral ischemia in rat. Stem Cells Transl. Med..

[16] Zhu Y (2008). Are TGF-beta1 and bFGF correlated with bladder underactivity induced by bladder outlet obstruction?. Urol. Int..

[17] Liang CC, Shaw SS, Chou HH, Huang YH, Lee TH (2020). Amniotic fluid stem cells improve rat bladder dysfunction after pelvic nerve transection. Cell Transplant..

[18] Ma FH, Higashira-Hoshi H, Itoh Y (2002). Functional muscarinic M2 and M3 receptors and beta-adrenoceptor in cultured rat bladder smooth muscle. Life Sci..

[19] Livak KJ, Schmittgen TD (2001). Analysis of relative gene expression data using real-time quantitative PCR and the 2(-Delta Delta C(T)) Method. Methods.

[20] Elbadawi A, Yalla SV, Resnick NM (1993). Structural basis of geriatric voiding dysfunction. IV. Bladder outlet obstruction. J. Urol..

[21] Deveaud CM (1998). Molecular analysis of collagens in bladder fibrosis. J. Urol..

[22] Lee HJ (2012). Inhibition of collagen deposit in obstructed rat bladder outlet by transplantation of superparamagnetic iron oxide-labeled human mesenchymal stem cells as monitored by molecular magnetic resonance imaging (MRI). Cell Transplant..

[23] Al-Saikan B, Ding J, Tredget E, Metcalfe P (2016). Benefits of mesenchymal stem cells after partial bladder outlet obstruction. Can. Urol. Assoc. J..

[24] Hughes FM, Sexton SJ, Jin H, Govada V, Purves JT (2017). Bladder fibrosis during outlet obstruction is triggered through the NLRP3 inflammasome and the production of IL-1beta. Am. J. Physiol. Renal. Physiol..

[25] Tanaka ST (2009). Recruitment of bone marrow derived cells to the bladder after bladder outlet obstruction. J. Urol..

[26] Wiafe, B. *et al.* Mesenchymal stem cell therapy inhibited inflammatory and profibrotic pathways induced by partial bladder outlet obstruction and prevented high-pressure urine storage. *J Pediatr Urol* **15**, 254 e251–254 e210, 10.1016/j.jpurol.2019.03.003 (2019). 10.1016/j.jpurol.2019.03.003 30967358

[27] Woo LL (2011). Mesenchymal stem cell recruitment and improved bladder function after bladder outlet obstruction: preliminary data. J. Urol..

[28] Sanchez-Elsner T (2001). Synergistic cooperation between hypoxia and transforming growth factor-beta pathways on human vascular endothelial growth factor gene expression. J. Biol. Chem..

[29] Jiang X (2015). Sodium Tanshinone IIA Sulfonate Ameliorates Bladder Fibrosis in a Rat Model of Partial Bladder Outlet Obstruction by Inhibiting the TGF-beta/Smad Pathway Activation. PLoS ONE.

[30] Haung D (2009). Defining the role of hif-1 ? And ctgf in fibrosis—biomed 2009. Biomed. Sci. Instrum..

[31] Chowdhury I, Chaqour B (2004). Regulation of connective tissue growth factor (CTGF/CCN2) gene transcription and mRNA stability in smooth muscle cells. Involvement of RhoA GTPase and p38 MAP kinase and sensitivity to actin dynamics. Eur. J. Biochem..

[32] Mezzano V, Cabrera D, Vial C, Brandan E (2007). Constitutively activated dystrophic muscle fibroblasts show a paradoxical response to TGF-beta and CTGF/CCN2. J. Cell Commun. Signal.

[33] Wiafe B, Kadam R, Metcalfe PD (2020). Intraperitoneal administration of mesenchymal stem cells is effective at mitigating detrusor deterioration after pBOO. Am. J. Physiol. Renal Physiol..

[34] Lutolf R (2018). NLRP3/IL-1beta mediates denervation during bladder outlet obstruction in rats. Neurourol. Urodyn..

[35] Song YS (2012). Mesenchymal stem cells overexpressing hepatocyte growth factor (HGF) inhibit collagen deposit and improve bladder function in rat model of bladder outlet obstruction. Cell Transplant..

[36] Bunt SK, Sinha P, Clements VK, Leips J, Ostrand-Rosenberg S (2006). Inflammation induces myeloid-derived suppressor cells that facilitate tumor progression. J. Immunol..

[37] Bunt SK (2007). Reduced inflammation in the tumor microenvironment delays the accumulation of myeloid-derived suppressor cells and limits tumor progression. Cancer Res..

[38] Lin WY (2014). Transient increase in circulating myeloid-derived suppressor cells after partial bladder outlet obstruction. J. Urol..

[39] Chermansky C, Mitsogiannis I, Abrams P, Apostolidis A (2019). Stem cells and lower urinary tract dysfunction: Has its potential finally reached clinical maturity? ICI-RS2018. Neurourol. Urodyn..

[40] Levanovich PE (2015). Intradetrusor injection of adult muscle-derived cells for the treatment of underactive bladder: Pilot study. Int. Urol. Nephrol..

[41] Diokno JPG, Sirls LT, Hasenau DL, Bowlus JE, Shea EM, Chancellor MB (2019). J. Urol..

[42] Liang CC, Shaw SS, Ko YS, Huang YH, Lee TH (2020). Effect of amniotic fluid stem cell transplantation on the recovery of bladder dysfunction in spinal cord-injured rats. Sci. Rep..

