# Supplementary material for: Human amniotic fluid stem cells can alleviate detrusor dysfunction caused by bladder outlet obstruction in rats
Source: Sci Rep. 2022 Apr 23;12:6679. doi: 10.1038/s41598-022-10640-y (PMC9035144; doi:10.1038/s41598-022-10640-y)
Supplement: Supplementary file 1 — Supplementary Information 1. [file 41598_2022_10640_MOESM1_ESM.pdf]

Human amniotic fluid stem cells can alleviate detrusor dysfunction caused by bladder outlet obstruction in rats

Ching-Chung Liang,[1,2] Wen-Chu Huang,[3,4] Steven W. Shaw,[2,5,6] Yung-Hsin Huang,[1] Tsong-Hai Lee[*,2,7]

[1]Female Urology Section, Department of Obstetrics and Gynecology, Chang Gung Memorial Hospital Linkou Medical Center, Taoyuan, Taiwan

[2]College of Medicine, Chang Gung University, Taoyuan, Taiwan

[3]Division of Urogynecology, Department of Obstetrics and Gynecology, Mackay Memorial Hospital, Taipei, Taiwan

[4]Department of Nursing, Mackay Junior College of Medicine, Nursing, and Management, Taipei, Taiwan

[5]Division of Obstetrics, Department of Obstetrics and Gynecology, Taipei Chang Gung Memorial Hospital, Taipei, Taiwan

[6]Prenatal Cell and Gene Therapy Group, Institute for Women's Health, University College London, London, UK

[7]Stroke Center and Department of Neurology, Chang Gung Memorial Hospital, Linkou Medical Center, Taoyuan, Taiwan

*Correspondence: Tsong-Hai Lee, MD, PhD
Stroke Center and Department of Neurology,
Chang Gung Memorial Hospital, Linkou Medical Center, Taoyuan, 333 Taiwan
No. 5, Fu-Hsing Street, Kweishan, Taoyuan, 333 Taiwan
TEL: +886-3-3281200, ext. 8340; FAX: 886-3-3288849;
E-mail:thlee@adm.cgmh.org.tw

Running title: stem cells alleviate detrusor dysfunction

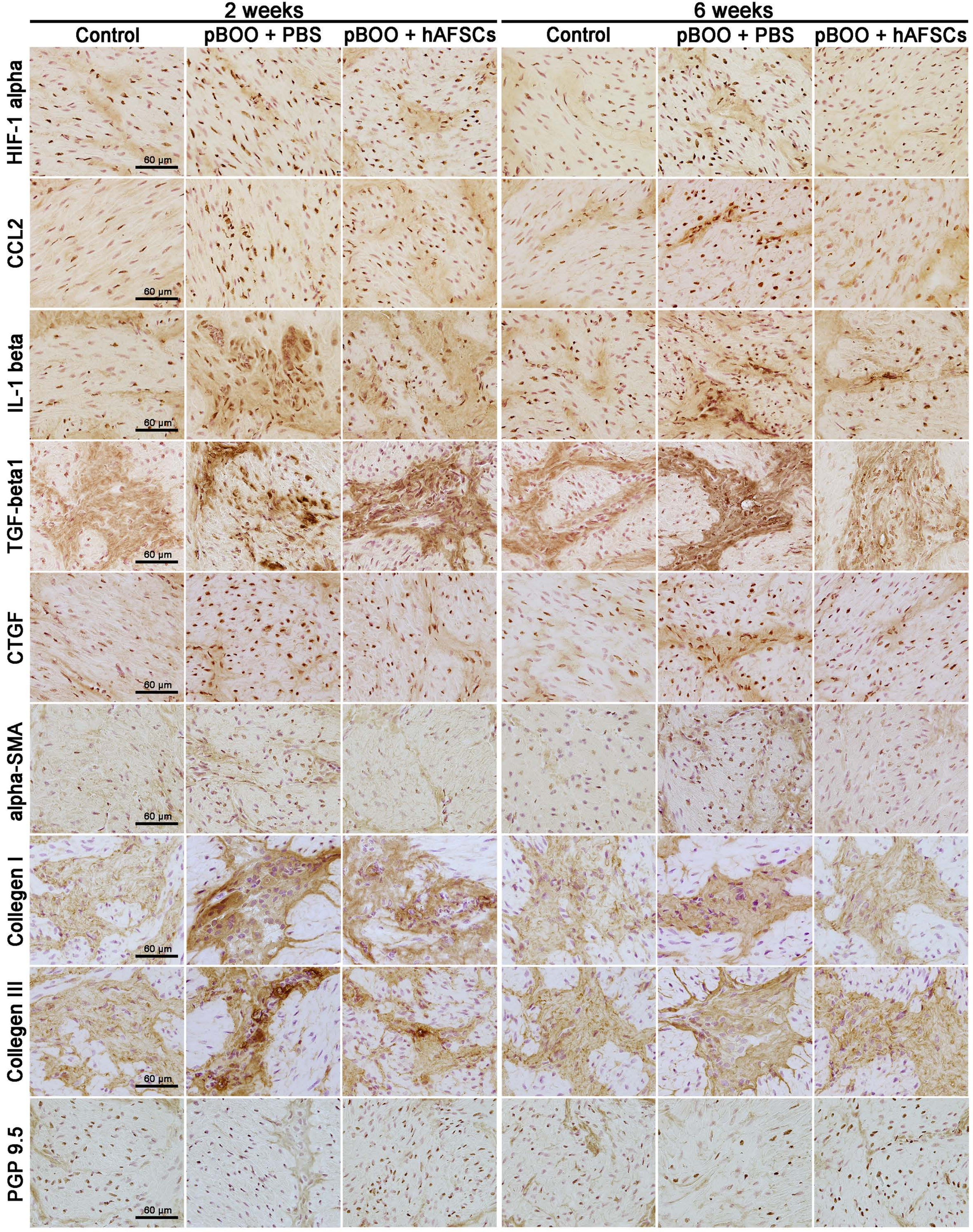
2 weeks
6 weeks
Control
pBOO + PBS
pBOO + hAFSCs
Control
pBOO + PBS
pBOO + hAFSCs
HIF-1 alpha
CCL2
IL-1 beta
TGF-beta1
CTGF
alpha-SMA
Collegen I
Collegen III
PGP 9.5
60 μm
