# Supplementary material for: Human amniotic fluid stem cells can alleviate detrusor dysfunction caused by bladder outlet obstruction in rats
Source: Sci Rep. 2022 Apr 23;12:6679. doi: 10.1038/s41598-022-10640-y (PMC9035144; doi:10.1038/s41598-022-10640-y)
Supplement: Supplementary file 2 — Supplementary Information 2. [file 41598_2022_10640_MOESM2_ESM.docx]

**Supplementary Table 1.** Primary antibodies used for the immunohistochemical investigations

| Primary antibody | Type, Catalogue number | Isotype | Dilution | Host species | Manufacturer |
| --- | --- | --- | --- | --- | --- |
| HIF1α | polyclonal, ab216842 | IgG | 1:250 | Rabbit | Abcam, Cambridge, USA |
| CCL2 | polyclonal, ab7202 | IgG | 1:250 | Rabbit | Abcam, Cambridge, USA |
| IL-1β | polyclonal, A16288 | IgG | 1:200 | Rabbit | ABclonal Biotechnology, USA |
| TGF-β1 | monoclonal, sc-130348 | IgG | 1:100 | Mouse | Santa Cruz Biotechnology, USA |
| CTGF | polyclonal, ab6992 | IgG | 1:250 | Rabbit | Abcam, Cambridge, USA |
| alpha-SMA | monoclonal, A2547 | IgG | 1:500 | Mouse | Sigma, St. Louis, MO, USA |
| Collagen I | monoclonal, ab6308 | IgG | 1:250 | Mouse | Abcam, Cambridge, USA |
| Collagen III | monoclonal, ab6310 | IgG | 1:250 | Mouse | Abcam, Cambridge, USA |
| PGP9.5 | monoclonal, ab8189 | IgG | 1:250 | Mouse | Abcam, Cambridge, USA |
| CD11b/c | monoclonal, ab1211 | IgG2a | 1:500 | Mouse | Abcam, Cambridge, USA |
| HIS48 | monoclonal, ab33760 | IgM | 1:50 | Mouse | Abcam, Cambridge, USA |

HIF1α = Hypoxia inducible factor-1α, IL-1β = Interleukin-1β, TGF-β1 = Transforming growth factor-β1, CTGF = Connective tissue growth factor, α-SMA = α-smooth muscle actin, PGP9.5 = Protein gene product 9.5.
