# Supplementary material for: Human amniotic fluid stem cells can alleviate detrusor dysfunction caused by bladder outlet obstruction in rats
Source: Sci Rep. 2022 Apr 23;12:6679. doi: 10.1038/s41598-022-10640-y (PMC9035144; doi:10.1038/s41598-022-10640-y)
Supplement: Supplementary file 3 — Supplementary Information 3. [file 41598_2022_10640_MOESM3_ESM.docx]

**Supplementary Fig. S1.**

Immunostaining of hypoxia inducible factor-1α (HIF1α), CCL2, interleukin-1β (IL-1β), transforming growth factor-β1 (TGF-β1), connective tissue growth factor (CTGF), α-smooth muscle actin (α-SMA), collagen I, collagen III and protein gene product 9.5 (PGP9.5) in the bladder of control, pBOO + PBS and pBOO + hAFSCs treatment. The statistical results are presented in Figure 5. pBOO = Partial bladder outlet obstruction, PBS = Phosphate-buffered saline, hAFSCs = Human amniotic fluid stem cells, PCR = Polymerase chain reaction, HIF1α = Hypoxia inducible factor-1α, IL-1β = Interleukin-1β, TGF-β1 = Transforming growth factor-β1, CTGF = Connective tissue growth factor, α-SMA = α-smooth muscle actin, PGP9.5 = Protein gene product 9.5. Bar indicates 60 μm.
